# Synthesis of Theaflavins and Their Functions

**DOI:** 10.3390/molecules23040918

**Published:** 2018-04-16

**Authors:** Masumi Takemoto, Hiroaki Takemoto

**Affiliations:** 1 School of Pharmaceutical Sciences, Ohu University, 31-1 Tomitamachi-Aza Misumido, Koriyama, Fukushima 963-8611, Japan; 2 School of Pharmaceutical Sciences, Kitasato University, 5-9-1 Shirokane, Minato-ku, Tokyo 108-8641, Japan; 3 Faculty of Pharmaceutical Sciences, Toho University, Miyama 2-2-1, Funabashi, Chiba 274-8510, Japan

**Keywords:** black tea, theaflavins, synthesis, physiological functions, polyphenol oxidase, peroxidase

## Abstract

Numerous epidemiological and interventional clinical studies have consistently reported that black tea is good for human health. The polyphenolic compound, theaflavin, and its galloyl esters (theaflavins) are the primary red pigments in black tea that possess several health benefits, including fat-reducing and glucose-lowering capabilities and lifestyle-related disease prevention related to anti-obesity, anticancer, anti-atherosclerotic, anti-inflammatory, antiviral, antibacterial, anti-osteoporotic, and anti-dental caries properties. These compounds are produced by key enzymes, such as polyphenol oxidase and peroxidase, from parent green tea catechins present in fresh green tea leaves during the production of black tea leaves or the fermentation of green tea. However, theaflavins are only present in low concentrations in black tea; thus, their extraction from black tea leaves at sufficient levels for use in medical studies has been difficult. To circumvent this issue, different procedures for the synthesis of theaflavins using chemical oxidizing reagents or enzymes have been studied; however, low yields have limited their utility. Recently, however, several biosynthetic methods have been developed for the mass production of theaflavins. Using these methods, the physiological functions of theaflavins in lifestyle-related diseases in mice and humans have also been studied. In this review, we present the synthesis of theaflavins and their health benefits.

## 1. Introduction

During tea production, tea is largely separated into non-fermented tea and fermented tea. Enzymes such as polyphenol oxidase and peroxidase are present in tea plant leaves, which, for non-fermented tea, are either steamed, boiled, microwaved or electrically heated to inactivate endogenous oxidases. Black tea leaves are tea leaves in which green tea catechins are oxidized by endogenous polyphenol oxidase or peroxidase during the fermentation process [1,2,3,4].

**Figure 1:**
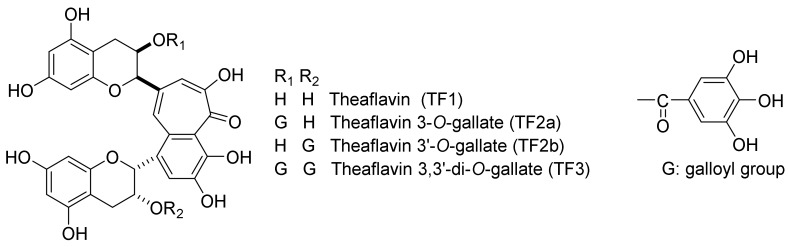
Chemical structures of TF1, TF2a, TF2b, and TF3.

In black tea leaves, the polyphenols are contained. Approximately 70 kinds of tea polyphenols with phenolic hydroxyl groups have been isolated, and many physiological functions have been reported. Tea polyphenols are roughly classified into the primary polyphenols contained in tea leaves and secondary polyphenols, which are converted from flavan-3-ols. Primary polyphenols are classified as flavan-3-ols, and hydrolyzable tannins. Secondary polyphenols include theaflavins, theaflagallins, and theasinensins. Theaflavin (TF1) and its galloyl esters represent the main red pigments in black tea; their chemical structures [TF1, TF 3-*O*-gallate (TF2a), TF 3′-*O*-gallate (TF2b), and TF 3,3′-di-*O*-gallate (TF3)] are shown in Figure 1.

The majority (75%) of the tea consumed all over the world is black tea. Both black tea and green tea are reportedly good for human health. For example, the consumption of black tea, which is rich in polyphenols, has been found to reduce ovarian cancer risk [5]. Indeed, TF1, TF2a, TF2b, and TF3 reduce the viability of ovarian cancer cells at lower concentrations than with normal ovarian cells. TF1 appears to mediate apoptosis via the intrinsic pathway, whereas the other theaflavins operate via both the intrinsic and extrinsic pathways. In addition, TF1 inhibits tube formation via reducing the secretion of vascular endothelial growth factor in a hypoxia-inducible factor 1α-independent manner, whereas the other theaflavins appear to work in a hypoxia-inducible factor 1α-dependent manner [5].

Furthermore, both epidemiological studies and interventional clinical studies have examined the relationship between drinking tea, such as green tea, oolong tea, black tea, and plasma cholesterol concentration, one of the major risk factors for arteriosclerosis. Indeed, Stensvold et al. have studied the relationship between the consumption of tea and serum cholesterol concentration in 9856 men and 10,233 women [all men and women were between 35 and 49 years of age and were from the county of Oppland (Norway)] without a history of cardiovascular disease or diabetes [6]. The mean serum cholesterol decreased among persons drinking more than five cups of tea compared with persons drinking no tea or drinking less than one cup. Green and Harari examined the relationship between serum total cholesterol concentration and amount of black tea or coffee drank in approximately 5300 Israeli factory employees without heart disease (about 4300 males and about 1700 females) [7]. Their findings indicated that when the consumption of coffee was five cups per day or more, the level of serum total cholesterol rose remarkably, whereas for black tea, serum total cholesterol levels decreased as consumption increased. Further studies are needed to evaluate the possible lipid-lowering effects of tea consumption.

In addition, cell-based and animal experiments using black tea extracts have been conducted to elucidate the functionality of black tea. In one study, the effects of Chinese green tea, oolong tea, and black tea on lipid metabolism were studied in young and mature rats fed a high fructose diet [8]. The high fructose diet induced dietary hyperlipidemia, but 4-week-old rats given black tea alone manifested a restrained elevation of lipids in the plasma. In groups of 8-week-old rats fed Chinese green tea, oolong tea, or black tea, levels of liver triglycerides and phospholipids decreased significantly. Further, Satoh et al. reported that black tea is effective against type 2 diabetes mellitus because it can help modulate postprandial hyperglycemia [9]. However, the precise mechanisms underlying the therapeutic and preventive effects on type 2 diabetes mellitus remain unclear. The authors demonstrated that the freeze-dried powder of the aqueous extract of black tea leaves (JAT) inhibited the degradation of disaccharides into monosaccharides by α-glucosidase in the small intestine. Half-maximal inhibitory concentration values also indicated that JAT significantly reduced α-glucosidase activity but weakly reduced α-amylase activity. These findings indicate that black tea could be useful as a functional food as part of a dietary strategy for borderline type 2 diabetes mellitus, resulting in the modulation of postprandial hyperglycemia. Furthermore, Kobayashi et al. reported that administration of black tea polyphenols at 100 and 200 mg/kg of body weight in rats suppressed postprandial hypertriacylglycerolemia in a dose-dependent manner [10]. TF3 was more effective in inhibiting the activity of pancreatic lipase than epigallocatechin gallate (EGCG), epicatechin gallate (ECG), or a mixture of EGCG and ECG. These results suggest that black tea polyphenols suppressed postprandial hypertriacylglycerolemia by reducing triacylglycerol absorption via the inhibition of pancreatic lipase activity.

Cancer of the prostate gland is the most common invasive malignancy and the second leading cause of cancer-related death in human males. However, many studies have shown that black tea reduces the risk of several types of cancer. Sun et al. studied the effects of active extracts of black tea and the TF black tea polyphenols on cellular proliferation and the mitochondria of the human prostate cancer cell line PC-3 [11]. Yinghong black tea extract (YBT), Assam black tea extract (ABT), and TFs inhibited cellular proliferation in a dose-dependent manner. In addition, TFs, YBT, and ABT affected the morphology of PC-3 cells and induced apoptosis, and sometimes necrosis, in PC-3 cells. These findings suggest that black tea may act as an effective antiproliferative agent in PC-3 cells. In these cells, TFs, YBT, and ABT appeared to induce apoptosis through mitochondrial dysfunction.

From the above reports, the physiological functionalities of black tea extracts have been studied all over the world. The major component of black tea extracts are TFs, including TF1, TF2a, TF2b, and TF3, which are the main red pigments in black tea (Figure 1). In recent years, TFs have attracted considerable interest, as they have been shown to have various physiological actions, including antioxidant [12], anticancer [13], anti-atherosclerotic [14], anti-inflammatory [15], antiviral [16,17,18], and anti-periodontitis [19,20] effects, as well as the prevention of osteoporosis [21,22]. Furthermore, these compounds have been shown to have human health benefits including glucose-lowering [23,24,25,26] and anti-obesity actions [10,27,28], as a prevention of lifestyle-related diseases.

Because of the extremely low concentration of TFs present in black tea, the extraction of TFs from black tea leaves in quantities sufficient for use in medical studies has been difficult. For these reasons, the synthesis of TFs by chemical oxidizing reagents or enzymatic methods has been reported; however, yields have been too low. The reasons for the low yield may be the polymerization of the composed TFs by long reaction times, post-treatment variables of the reaction or operation of the particular column.

Recently, however, several biosynthetic methods suitable for the mass production of TFs have been reported [26,28,29,30]. Furthermore, a method for producing TFs at the food manufacturing stage has been developed [31,32,33,34,35]. Takemoto et al. have reported TF1-containing fermentation water in obese mice on a high-fat diet inhibited body weight gain, decreased casual blood glucose and fasting blood glucose levels, and lowered mesenteric and total fat composition [26,36]. Furthermore, the authors reported the effect of this water on blood glucose levels in healthy humans and found that it significantly inhibited blood glucose levels [26].

Numerous reports have indicated that drinking black tea is good for health. Regarding the functionality, it is reported that TFs are higher than green tea catechins [10,23,26]. As mentioned, several biosynthetic methods of TFs suitable for mass production have been reported [26,28,29,30,32,36,37]. Using these methods, the physiological functions of TFs in mice [26,28,35] and humans [26] for the prevention of lifestyle-related diseases have recently been studied. In future studies, the detailed functionalities of TFs will likely be reported all over the world by using more precise animal experiments and human intervention tests. In this review, we introduce the synthesis of TFs and the functionality of theaflavins (antimetabolic syndrome, anti-periodontitis, anti-norovirus, anti-osteoporosis). 

## 2. Biosynthetic Pathway of TFs

TFs are compounds known to comprise the red pigment of black tea leaves. Roberts et al. separated red pigments from black tea products and gave them names such as theaflavin and thearubigin. TFs include TF1, TF2a, TF2b, and TF3 (Figure 1). It is known that TFs account for 2–6% in the dry weight of solids in brewed black tea approximately [4].

**Figure 2:**
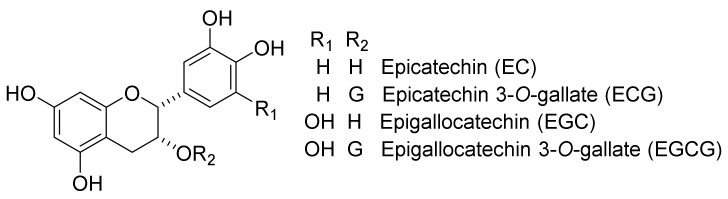
Chemical structures of EC, ECG, EGC, and EGCG.

TFs possess a benzotropolone skeleton. These compounds are produced from their parent catechins (Figure 2) [epicatechin (EC), ECG, epigallocatechin (EGC), and EGCG] by polyphenol oxidase (PPO) or peroxidase (POD) in fresh green tea leaves during the production of black tea leaves or green tea fermentation [1,2,3,4].

**Figure 3:**
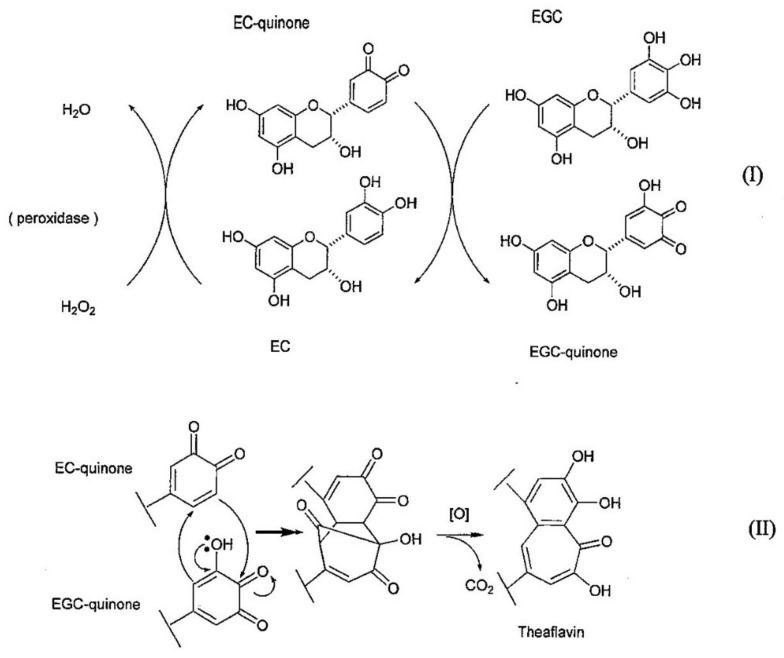
The biosynthetic pathway of TF1.

The biosynthetic pathway of TF1 has been reported. The biosynthetic pathway of TF1 from EC and EGC is shown in Figure 3 [1,38]. In this process, EC is first oxidized by polyphenol oxidase or peroxidase to EC-quinone; EC-quinone then oxidizes EGC to produce EGC-quinone. Next, Michael addition of the EGC-quinone to the EC-quinone occurs, followed by carbonyl addition, which produces a 3-membered ring intermediately. Subsequently, by oxidation and decarboxylation, the benzotropolone skeleton is formed and TF1 is generated.

**Figure 4:**
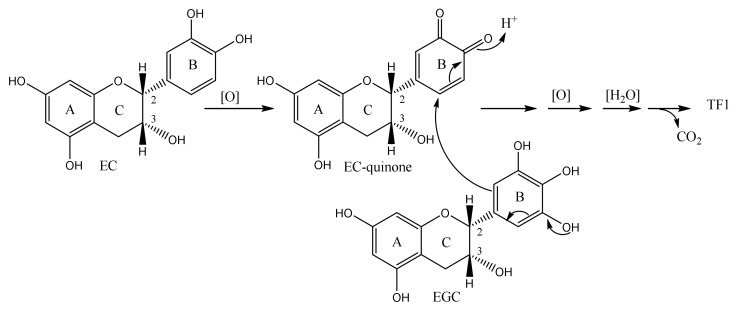
The biosynthetic pathway of TF1 by Nakatsuka et al.

Recently, Nakatsuka et al. reported the new biosynthetic pathway of TF1 (Figure 4) [39]. They reported the first step of TF1 was indicated as follows: first, EC become EC-quinone by the oxidation, next, EGC nucleophilic attacked to it.

## 3. Methods for Obtaining TFs and Relevant Obstacles

TFs are produced by enzymes in the leaves of *Camellia sinensis* by fermentation during the process of black tea production. In particular, green tea catechins [EC, ECG, EGC, and EGCG] in *C. sinensis* leaves, under the action of enzymes (particularly polyphenol oxidase and peroxidase), are oxidized or polymerized. In the process of black tea production, many of the flavan-3-ols become proanthocyanidin polymers, with small amounts then becoming TFs, theaflagallins, or theasinensins.

In the manufacturing process of black tea, the content of TFs is extremely low. For example, only about 1% of TFs by weight in total is contained in black tea. Takino et al. quantitated the compounds in black tea by Robert's method [40]. They reported that TFs decrease with longer fermentation times, but thearubigin increases significantly with longer fermentation. Thus, it is considered that the fact that the amount of TF in tea is small is attributed to fermentation time.

Physiological functionalities of black tea have been evaluated by epidemiological surveys, with the consumption of polyphenol-rich tea in cells or animal experiments using black tea extracts. TFs for these experiments were generally obtained by their extraction from black tea. Because of the extremely low concentration of TFs present in black tea, their extraction from black tea leaves in quantities sufficient for use in medical studies have been difficult. For these reasons, many synthetic methods of TFs by chemical oxidizing reagents or enzyme have been studied.

## 4. Conventional Synthetic Methods of TFs

Four types of TFs can be obtained by polyphenol oxidase or peroxidase in *C. sinensis* tea leaves with the combination of green tea catechins, as shown below:

① EC + EGC → TF1
② EC + EGCG → TF2a
③ ECG + EGC → TF2b
④ ECG + EGCG → TF3

To synthesize TF1, green tea catechin and a chemical oxidizing agent have been used. Takino et al. reported the synthesis of TF1 (crystalline reddish orange needles) using a chemical oxidizing reagent (potassium ferricyanide) for the first time in the world [38]. To the aqueous solution of EC (20 g) and EGC (10 g), potassium ferricyanide and sodium hydrogen carbonate were added to afford reddish orange needles of TF1 (1.6 g) by recrystallization from water and methanol. Collier et al. also reported the synthesis of TFs by ferricyanide oxidation [41]. Various TFs were synthesized using EGCG + EC, EGC + ECG, EGCG + ECG, gallic acid + EC, gallic acid + ECG, gallic acid + C, EGC + EC, and EGC + C as catechin combinations. The structures of these combinations were established by nuclear magnetic resonance and mass spectrometry.

After these studies, TF synthesis with oxidase became the mainstream. Polyphenol oxidase and peroxidase are present in fresh *C. sinensis* tea leaves. Polyphenol oxidase catalyzes the oxidation of substrates in the presence of oxygen, whereas peroxidase catalyzes the oxidation of substrates in the presence of hydrogen peroxide. Robertson et al. focused on polyphenol oxidase as an oxidase in *C. sinensis* leaves [42]. They studied an in vitro model fermentation system, containing purified catechins and partially purified polyphenol oxidase from green tea shoots under pure O_2_. Fermentation of a catechin mixture, containing the four major catechins, EC, EGC, EGCG, and ECG, at equal individual concentrations (55 mM), produced total TF1 levels 68% higher and thearubigin levels only 25% higher after 30 min than those from a standard catechin mixture fermented under similar conditions. Continued fermentation of this mixture produced no further TF1, but the thearubigin fraction increased. Next, Robertson studied TF1 and oxygen concentration during fermentation [43]. They reported low oxygen concentration during fermentation, as a result of inadequate aeration or high enzyme concentration, which was enhanced by high temperatures, resulting in the inhibition of theaflavin and the promotion of thearubigin production.

Margaret et al. also studied the stability of TFs obtained by the fermentation of tea leaves using polyphenol oxidase and peroxidase present in tea leaves [44]. A mixture of TFs was stable in the presence of air and tea polyphenoloxidase but decomposed rapidly to yield polymeric materials under the action of hydrogen peroxide and peroxidase derived from tea or horseradish. Furthermore, the authors suggested the influence of pH on the stability of TF.

Sinkar et al. used polyphenol oxidase and peroxidase to compare the content of TFs at pH 4.5 and pH 5.0 [45]. They also carried out in vitro oxidation experiments using PPO from fresh tea leaves, horseradish peroxidase (POD), and tea catechins as precursors for TFs. In vitro oxidation experiments using crude tea PPO resulted in a higher content of TFs at pH 4.5 in comparison with pH 5.5, which is the normal pH of macerated tea leaves. When purified PPO was used in the in vitro system, surprisingly, a reversal of this trend was observed, with more TFs being formed at the higher pH.

Next, some synthesis methods of TFs using peroxidase will be introduced. Sang et al. showed that horseradish POD can oxidize tea catechins to form TF-type compounds in the presence of H_2_O_2_ [46]. Furthermore, the authors reported the synthesis of 18 theaflavin derivatives using the horseradish POD/H_2_O_2_ system [47]. They synthesized benzotropolone derivatives, including TF1, TF2a, TF2b, TF3, neotheaflavin, neo TF-3-gallate, theaflavate A, theaflavate B, neotheaflavate B, theaflavic acid, epitheaflavic acid, epitheaflavic acid-3′-gallate, and epitheaflagallin 3-gallate, by using the horseradish POD/H_2_O_2_ system. For example, TF1 was obtained at 250 mg from EC (1.0 g) and EGC (1.0 g). This method may allow for the preparation of large quantities of pure theaflavin for biological assays.

Takemoto et al. reported a method using *C*. *sinensis* cell cultures for the efficient synthesis of TF1 from EC and EGC with high yields [25,48]. Horseradish peroxidase (HRP) is a commercially available metalloporphyrin enzyme. The authors observed that *C. sinensis* cell cultures are a rich source of peroxidase (POD) enzymes [49]. They surveyed a variety of oxidizing agents [commercial polyphenol oxidase (PPO) (Funakoshi Co., Ltd., Tokyo, Japan), commercial HRP (FUJIFILM Wako Co., Ltd. Osaka, Japan), *C. sinensis* cell cultures, *Nicotiana tabacum* cell cultures, and *Daucus carota* cell cultures] to optimize conditions of the synthesis of TF1 by the oxidation of EC and EGC [25,48]. EC (290 mg) and EGC (306 mg) were added to a mixture of acetone and phosphate buffer (pH 6.0) (1:10 *v*/*v*, 100 mL), *C. sinensis* cell culture (50 mL including 10.2 g cells), and 3% H_2_O_2_ (0.8 mL). The mixture was stirred for 4 min to provide TF1 (395 mg) with a 70% yield and 100% conversion. Next, Takemoto et al. used HRP, PPO, or *C. sinensis* cell cultures to synthesize TF2a from EC and EGCG, TF2b from ECG and EGC, and TF3 from ECG and EGCG. With respect to the yields of all TF2a, TF2b, and TF3, *C. sinensis* cell cultures were not superior to PPO or HRP. These results suggest that *C. sinensis* cell cultures contain specific enzymes for the synthesis of TF1 from EC and EGC.

Tanaka et al. synthesized TF1 using a plant homogenate not containing catechins [1]. They examined the production of TF1 from a mixture of EC and EGC by treatment with various plant homogenates and found that several plants are capable of synthesizing TF1, regardless of whether the plant contains catechins. The yields of TF1 on treatment with Japanese pear and loquat homogenates were much higher than that of fresh tea leaves. The addition of these plant homogenates into a green tea infusion produced TFs, indicating that the black tea pigments can be produced from green tea without any chemical reagents.

Recently, Nakatsuka et al. reported the new biosynthetic pathway of TF1 (Figure 4) [39]. By Nakatsuka’s pathway, new methods for non-enzymatic biomimetic synthesis of TFs were reported. Nakatsuka et al. reported an efficient synthesis of the 8,9-dihydroxybenzotropolone as a model compound of TFs by adding pyrogallol after oxidation of catechol by the treatment of Fetizon reagent (Ag_2_CO_3_/Celite) in over 70% yield [38]. Kan et al. reported biomimetic synthesis of TFs from catechins was accomplished by using 2-nitrobenzenesulfonyl (Ns) as a protecting group for phenols to minimize undesired side reactions of the electron-rich aromatic rings. This enabled the construction of the complex benzotropolone core in a single-step oxidative coupling reaction [50,51]. Matsuo and Tanaka et al. reported synthesis of TF1 using the DPPH radical as an oxidizing agent [52]. 

## 5. Biosynthetic Methods for the Mass Production of Theaflavins

The polyphenolic compound TF1 is reported to elicit various physiological effects. Similar to other theaflavins, the extremely low concentration of TF1 present in black tea makes its extraction from black tea leaves in sufficient quantities difficult. Next, the mass production of TF1 will be introduced.

**Figure 5:**
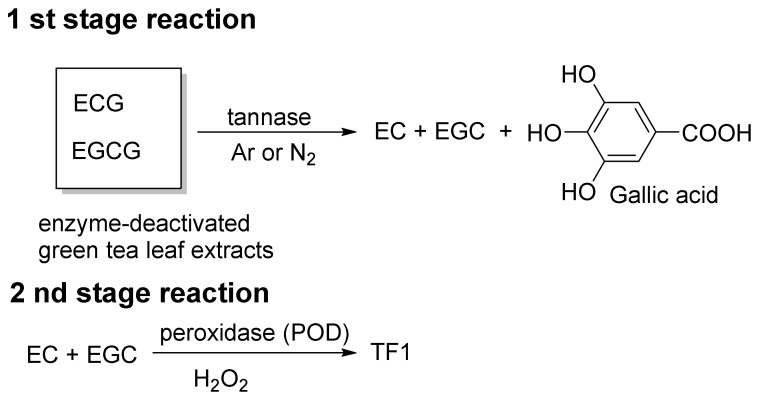
The William method of TF1 synthesis.

William et al. reported the mass production of TF1 [36]. In their method, a slurry of tea plant leaves was treated with tannase, and the slurry was fermented. Leaves are removed from the slurry to provide TF1-rich tea liquor (tea liquor) and separated tea leaves (doll). The tea liquor and doll are both rich in TF1. Their method includes a two-stage reaction (Figure 5). In the first stage of the reaction, the parent catechins EC, ECG, EGC, and EGCG, present in enzyme-deactivated green tea leaf extracts, are converted to EC, EGC, and gallic acid by exogenous tannase under argon or nitrogen. In other words, the gallate groups of ECG and EGCG are cleaved by the tannase to produce EC, EGC, and gallic acid (Figure 5, first stage reaction). In the second stage of the reaction, EC and EGC generated by the first reaction are converted to TF1 by exogenous POD and H_2_O_2_.

Takemoto et al. developed a simple and inexpensive mass production method of TF1 that is suitable for use in medical studies [28,29]. After soaking enzyme-deactivated dry Japanese green tea leaves (75 g) in water (1500 mL) for 12 h, the water mixture was separated by filtration to give the resulting tea-leaf filtrate (1840 mL), which contained 488.5 mg of EC, 122.4 mg of ECG, 556.5 mg of EGC, 610.5 mg of EGCG, gallic acid, and 302.1 mg of caffeine. Next, *C. sinensis* cell cultures (42 g of cells and 200 mL of broth) and 14.5 mL of 3% H_2_O_2_ were added to the enzyme-deactivated dry green tea-leaf filtrate (1840 mL), and the mixture was shaken at 110 rpm in air for 8 h to yield TF1 (193 mg).

**Figure 6:**
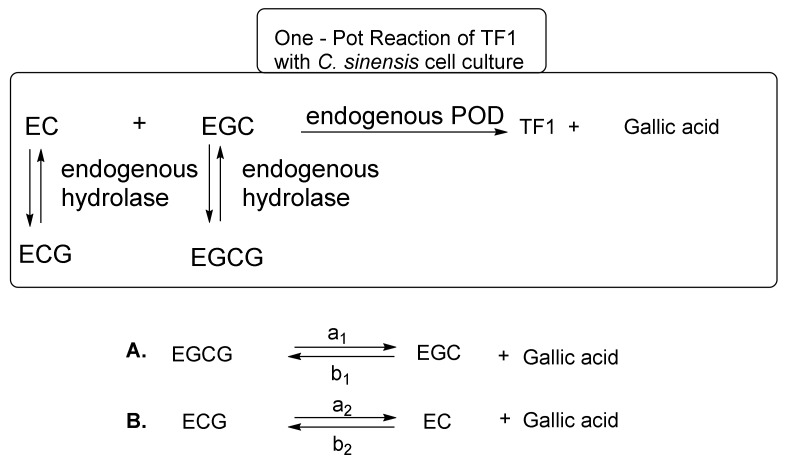
Equilibrium of EGCG, EGC, ECG, EC, and gallic acid.

These reaction mechanisms are shown in Figure 6. *C. sinensis* cell cultures are a superior system for TF1 synthesis from EC and EGC. However, *C. sinensis* cell cultures did not effectively synthesize TF2a from EC and EGCG, TF2b from ECG and EGC, or TF3 from ECG and EGCG [25]. In the one-pot reaction, EC and EGC are immediately converted to TF1 by endogenous POD. When the amount of EC and EGC in the reaction mixture has sufficiently decreased, the *C. sinensis* cell culture hydrolase initiates hydrolysis of ECG and EGCG to produce EC, EGC, and gallic acid. Importantly, the hydrolysis reaction is an equilibrium reaction (Figure 6). It is thought that the hydrolysis reaction of EGCG and ECG progressed to make up for EC and EGC in the reaction mixture immediately after TF1 generation from EC and EGC progressed. The EC and EGC generated by the hydrolysis reaction, in turn, is converted to TF1 by POD. Thus, TF1 synthesis and the hydrolysis of ECG and EGCG are repeated to yield TF1 and gallic acid from EC, EGC, ECG, and EGCG in the one-pot reaction. This new TF1 production method does not require the use of exogenous tannase, exogenous POD, nitrogen, or argon, making it completely different from the method described by William et al. [36]. Furthermore, all the necessary enzymes are present in the *C. sinensis* cell culture. However, this method was necessary to prepare catechins [EC, EGC, ECG, and EGCG] as raw materials, because *C*. *sinensis* cell cultures do not contain the four kinds of catechins.

Takemoto et al. focused their efforts on fresh tea plant leaves or frozen fresh tea plant leaves containing the four major *epi*-type catechins [EC, ECG, EGC, and EGCG] and enzymes (PPO, POD, and hydrolase) [26,31]. However, the enzymatic makeup in fresh or frozen tea plant leaves varies greatly from that in *C. sinensis* cell cultures. Particularly, *C. sinensis* cell cultures have POD and hydrolase, whereas fresh or frozen fresh tea plant leaves have PPO, POD, and hydrolase. When POD and PPO exist, PPO predominates. Takemoto et al. developed an easy method to control PPO and POD in frozen, fresh tea plant leaves. PPO catalyzes the oxidation of substrates in the presence of oxygen. On the other hand, POD catalyzes the oxidation of substrates in the presence of hydrogen peroxide. Oxygen-blocking methods can inactivate PPO. The use of an argon atmosphere is a common method; however, this method is expensive and difficult to perform. To create a more cost-effective method, they pulverized fresh, frozen tea plant leaves in the presence of a large quantity of water with a mixer for 1 min at 25 °C and then allowed the mixture to stand at 25 °C. In the stationary method, PPO activity decreased as dissolved oxygen in the water decreased. When they used 80 volumes of water to the weight of frozen, fresh tea plant leaves (10 g), pulverized for 1 min at 25 °C, and allowed the mixture to stand for 120 h at 25 °C, TF1 (85 mg) was also obtained as the sole product. Next, they investigated methods to shorten the reaction time to only 40 min, and TF1 could be successfully obtained by using approximately 480 g of frozen, fresh green tea leaves pulverized for 1 min in the presence of 50 volumes of water, with stirring at low speeds to minimize the introduction of air to afford TF1 (1.7 g). In their semi-anaerobic stirring method, the one-pot domino-type enzymatic selective reaction progressed to obtain TF1 as shown in Figure 6. The advantages of this method are that it is simple and inexpensive, and it provides the means to a large-scale production of TF1, which is vital for relevant physiological studies in mice and humans.

## 6. Manufacturing Methods of Foods Containing TFs

In this section, we introduce manufacturing methods of foods containing TFs. As mentioned in Section 5, because the TF content in black tea is low, it is costly to use black tea as a raw material for foods containing TF.

Sinija et al. reported a novel technology to produce instant/soluble tea powder from the expressed juice of green leaves [37]. Plucked leaves were crushed to a fine paste in a domestic mixer grinder, and about 320 mL of juice and 566 g of residue were obtained from 1 kg of green leaves. Instant tea was made from the fermentation of juice in the presence of oxygen for 1 h. Tea granules were made from the fermentation of the pressed leaf residue for 1.5 h. Instant tea included theaflavin (0.915%) and thearubigin (9.88%). Tea granules included TFs (0.566%) and thearubigin (6.86%).

Takemoto et al. developed a method that produces TFs at the food manufacturing stage.

i. Manufacturing method of a fermented tea beverage rich in TF1.

Takemoto et al. patented a manufacturing method for a fermented tea rich in TF1 [31]. Water (25 L) was added to 480 g of frozen tea plant leaves, and the mixture was pulverized with an industrial mixer for 1 min at 25 °C. The mixture was then stirred with an industrial stirrer at low speed (300 rpm) at 25 °C to prevent the contamination of air for 40 min. The mixture was filtered and retort sterilized to afford fermented tea rich in TF1 (3.5 g) and gallic acid (5.0 g). This patented method is based on the enzymatic selective synthesis of TF1 from EC, ECG, EGC, and EGCG [26,30].

ii. Preparation method of a fermented tea beverage rich in TFs.

Takemoto et al. patented a preparation method of a fermented tea beverage rich in TFs [32]. This patented method is based on the enzymatic synthesis of TFs from green tea catechins. Water (25 L) was added to 306 g of frozen tea plant leaves, and the mixture was pulverized with an industrial mixer for 3 min at 25 °C. The mixture was then shaken for 40 min, filtered, and retort sterilized to afford a fermented tea rich in TFs, including TF1 (1.9 g), TF2 (1.2 g), TF3 (0.8 g), and TF4 (1.1 g).

iii. Method for producing a fermented tea beverage containing TF1 and methylated catechin.

“Benifuuki” is a tea cultivar that contains methylated catechins such as epigallocatechin-3-*O*-(3-*O*-methyl) gallate (EGCG3″Me), which have anti-allergic actions. EGCG3″Me is extremely bitter and disappears by fermentation during the process of black tea production. In the case of “Benifuuki”, the green tea production method is recommended [53].

Takemoto et al. patented a method for producing a fermented tea beverage containing TF1 and EGCG3″Me [33]. Water (900 mL) was added to 100 g of frozen “Benifuuki” leaves, and the mixture was pulverized with an industrial mixer for 1 min at 25 °C. The mixture was allowed to stand for 24 h. The mixture was then filtered and retort sterilized to afford a fermented tea with TF1 (186 mg), TF2a (45.6 mg), TF2b (45.5 mg), TF3 (19.2 mg), EGCG (3.0 g), EGC (126 mg), and EGCG3″Me (104 mg). They succeeded in producing a fermented “Benifuuki” black tea beverage with TFs without losing EGCG3″Me. This fermented “Benifuuki” black tea beverage reportedly has an excellent taste and aroma despite containing EGCG3″Me.

iv. Production method of a tea powder containing TFs.

Takemoto et al. patented a production method for a tea powder containing TFs [35]. They added 5 L of water to 200 g of frozen green tea leaves and pulverized them with an industrial mixer for 1 min at 25 °C. The mixture was stirred with an industrial stirrer at low speed (300 rpm) at 25 °C to prevent the contamination of air for 40 min. The mixture was filtered, steamed, centrifuged, and spray-dried to obtain a tea powder containing TF1.

In a second method, they added 25 L of water to 306 g of frozen tea plant leaves and pulverized them with an industrial mixer for 3 min at 25 °C. The mixture was shaken for 40 min. The mixture was then filtered, steamed, centrifuged, and spray-dried to obtain a tea powder containing TF1, TF2a, TF2b, and TF3.

Specifically, the tea powder containing TFs (Yokoyama Food Co., Ltd., Sapporo, Japan) has been shown to be useful in animal studies as a prevention and improvement treatment for lifestyle diseases [30,35].

## 7. Manufacturing Methods for Grain Flour Processed Foods Containing TFs

Breads, noodles, and cookies made from cereal flour by adding black tea are commercially available. Because the price of the black tea leaves containing TFs is very high, the black tea used for these types of food production contains almost no TFs. However, Takemoto et al. patented a manufacturing method for grain flour processing foods (various kinds of foods such as bread, noodles, pasta, cookies, biscuits, cakes, dumplings and Shumai's leather, pizza, and nan peels, and manju skin) containing TF1 or TFs [34]. They developed a method to convert catechins into TFs with raw green tea leaves at the stage of the production of grain flour processed food. In particular, these foods showed superior rich texture as compared with foods made by using no raw green tea leaves. In the case of fermenting pulverized raw green tea leaves in water, the methods are different if we need to ① produce TF1 selectivity, ② produce four kinds of TFs, or ③ produce TF1 and methylated catechin. However, when cereal flour is used instead of water, we can freely manufacture ①–③ using the same method. For example, the manufacturing method of breads is described. Bread is produced by preparing dough from wheat flour, sugar, salt, butter, and green tea leaves (green tea leaves are pulverized in water with mixer) using baker’s yeast, fermenting, and baking the dough. In the case of ①, green tea leaves picked in June are used. In the case of ②, green tea leaves picked in April are used. In the case of ③, “Benifuuki” is used.

## 8. Health Benefits of TFs

### 8.1. Antimetabolic Syndrome

The main cause of metabolic syndrome is obesity, and cases of fatty liver, hypertension, and hyperlipidemia diseases caused by obesity are increasing year by year. Consequently, diabetes and arteriosclerosis can result. The suppression of obesity through the suppression of fat absorption by green tea or green tea ingredients has been suggested [54,55]. On the other hand, several meta-analyses have shown that the consumption of black tea results in significant primary prevention of cardiovascular diseases by decreasing plasma low-density lipoprotein cholesterol levels [56] and blood pressure [57]. It has also been reported that the consumption of black tea is inversely associated with body mass index [58]. Although it is known that black tea represents 78% of tea production worldwide [59], there has been only limited research on the biological significance of the ingestion of theaflavins.

Kobayashi et al. investigated the effects of black tea polyphenols on postprandial hypertriacylglycerolemia using rats [10]. The administration of black tea polyphenols at 100 and 200 mg/kg of body weight in rats suppressed postprandial hypertriacylglycerolemia in a dose-dependent manner. Furthermore, black tea polyphenols dose-dependently inhibited the activity of pancreatic lipase in vitro with an IC_50_ of 0.254 mg/mL. When purified TFs, which are components of black tea polyphenols, were used, TFs with galloyl moieties, but not those without the galloyl moiety, inhibited the activity of pancreatic lipase. TF3 was more effective in inhibiting the activity of pancreatic lipase than EGCG, ECG, or a mixture of EGCG and ECG. Black tea polyphenols and TF3 had a similar effect in inhibiting the activity of pancreatic lipase when the total polyphenol amount was adjusted to be the same. These results suggest that black tea polyphenols suppress postprandial hypertriacylglycerolemia by reducing triacylglycerol absorption via the inhibition of pancreatic lipase activity.

Furthermore, Kudo et al. examined the effect on energy expenditure of administering a single oral dose of a theaflavin-rich fraction. The authors used indirect calorimetry and monitored the initial metabolic changes in skeletal muscle and brown adipose tissue in mice [27]. Oxygen consumption (VO_2_) and energy expenditure were increased significantly in mice treated with the TF1-rich fraction (10 mg/kg) compared with the group administered vehicle alone, without a difference in locomotor activity. Furthermore, the mRNA levels of uncoupling protein (UCP)-1 and peroxisome proliferator-activated receptor gamma coactivator-1a (PGC-1α) in brown adipose tissue were increased significantly 2 h after administration of the TF1-rich fraction. The levels of UCP-3 and PGC-1α in the gastrocnemius muscle were also increased significantly 2 and 5 h after administration of the TF1-rich fraction. The concentration of phosphorylated AMP-activated protein kinase 1α was also increased significantly in the gastrocnemius 2 and 5 h after treatment with the TF1-rich fraction. These results indicate that the TF1-rich fraction significantly enhanced the systemic energy expenditure, as evidenced by an increase in the expression of metabolic genes. 

Diabetes mellitus, due to the worsening of lifestyle, generally refers to type 2 diabetes. Type 2 diabetes is a chronic disease caused by an inherited and/or acquired deficiency in insulin secretion and/or by decreased responsiveness of the organs to secreted insulin (insulin resistance). Such a deficiency results in increased blood glucose levels, which in turn can damage many of the body's systems, including blood vessels and nerves [60]. One successful approach to achieve optimal blood glucose levels is through the retardation of α-glucosidase activity [60], in a method similar to that of acarbose in the small intestine [61]. Green tea or the green tea ingredient, EGCG, possesses α-amylase and α-glucosidase inhibitory effects, and it has been reported that the daily consumption of one or more cups of black tea reduces the risk of diabetes mellitus by 14% compared with no consumption [62]. In addition, Satoh et al. reported that the half maximal inhibitory concentration values indicated that black tea extract significantly reduced α-glucosidase activity but weakly reduced α-amylase activity [9]. To clarify the active components in black tea showing the postprandial glucose suppression effect, Matsui T. et al. examined the inhibitory effects of catechins and TFs against α-glucosidase [23]. It was initially demonstrated that theaflavins and catechins preferentially inhibited maltase, rather than sucrase, in an immobilized α-glucosidase inhibitory assay system. For the maltase inhibitory effects of TFs, effects were observed in descending order of potency of TF2a > TF3 > TF2b > TF1. Furthermore, TF2a was the most potent maltase inhibitor among the various TFs and catechins. This suggests that the α-glucosidase inhibition induced by TFs is closely associated with the presence of a free hydroxyl group at the 3′-position of TF, as well as the esterification of TF with a mono-gallate group. In addition, the *R*-configuration at the 3′-position of TF2a showed a higher inhibitory activity compared with the *S*-configuration. As a result of a single oral administration of maltose (2 g/kg) in rats, a significant reduction in blood glucose level was observed at a dose of 10 mg/kg of TF2a. TF2a can suppress glucose production from maltose through the inhibition of α-glucosidase in the gut.

These results suggest that supplementing the diet with TFs may alter glucose availability and metabolism. However, the in vivo response of serum glucose to feeding TFs remains elusive despite the relevance to individuals with hyperglycemia. Therefore, Miyata et al. investigated how TFs affect serum glucose levels in the KK-A^y^ mouse, a model for type 2 diabetes, and serum and hepatic triacylglycerol concentrations in Sprague−Dawley rats fed a high-fat diet [24]. Feeding male KK-A^y^ mice diets with 0.1% TFs for 6 weeks reduced serum glucose levels by >30% compared with a control diet. Rats fed diets containing 0.2% TFs for 4 weeks had higher fecal fat excretion and 33% lower hepatic triacylglycerol; hepatic fatty acid synthase activity was not affected. Oral administration of TFs reduced the increase in serum triacylglycerol after an oral bolus of a fat emulsion. These results indicate that theaflavins induce antihyperglycemic responses in diabetic mice and are hypotriacylglycerolemic in rats by suppressing intestinal fat absorption.

Takemoto et al. reported the long-term anti-obesity effects of synthetic TF1 on lifestyle-related disease in 6-week-old male C57BL6 mice given a high-fat diet for 12 weeks [28]. Oral administration of TF1 (1.3 mg/day) for 12 weeks significantly inhibited both body weight gain and visceral fat accumulation [Body weight (g) control 38.7 ± 2.8, TF1: 36.2 ± 2.7 *, Abdomen length (mm) control 103 ± 3.1, TF1: 96.0 ± 6.14 **, Total fat (g) control 6.81 ± 0.98, TF1: 5.85 ± 0.91 *, perirenal fat (g) control 0.98 ± 0.12, TF1: 0.80 ± 0.20 *, perigluteal fat (g) control 2.05 ± 0.21, TF1: 1.50 ± 0.44 *] with no significant difference observed in the amount of feces between the experimental and control mice.

Takemoto et al. reported that oral administration of synthetic TF1 (30.6 mg/kg) to 5-week-old C57BL6 mice (*n* = 6) significantly inhibited increases in blood glucose in a sucrose loading test (2 g/kg) relative to the effect of the administration of distilled water in control mice (maximum blood sucrose levels 20 min after administration of TF: 195.0 ± 22.4 *, control: 220.0 ± 8.2) [49]. Five-week-old C57BL6 mice were provided with TF1 *ad libitum* (5 mg/15 mL water) and a high-fat diet, Quick Fat. When the mice (*n* = 7) were 16-weeks-old, sucrose (2 g/kg) or glucose (2 g/kg) was administered. Increases in blood glucose levels were significantly inhibited relative to those in the control group of mice that received distilled water (blood sucrose levels 40 min after sucrose administration of TF1: 234.0 ± 19.8 **, control: 309.3 ± 27.0; blood glucose levels 20 min after glucose administration of TF1: 407.6 ± 35.6 *, control: 482.6 ± 17.2).

Takemoto et al. reported the physiological function in mice and humans with TF1-containing fermentation water. Five-week-old healthy C57BL6 male mice were provided with either control water, TF1-containing fermentation water (4 mg/100 mL), a commercial black tea beverage, or a commercial green tea beverage; all groups were fed liquid consumption (2 mL per day) and a high-fat diet (HFD 32, 3.4 g per day) *ad libitum* for 40 weeks [26]. TF1-containing fermentation water significantly decreased fasting blood glucose levels in 36- or 40-week-old mice (40 weeks of age, control 165.2 ± 5.9, black tea 158.8 ± 8.4, green tea 156.3 ± 7.3, TF1-water 136.0 ± 5.0 *). Furthermore, TF1-containing fermentation water significantly decreased the rate of body weight gain based on the body weight at 5 weeks of age (16 weeks age, control 174 ± 3.6, black tea 171 ± 5.5, green tea 164 ± 2.6, TF-water 160 ± 2.7 *) [26]. At 40 weeks of age, mesenteric fat and total fat were significantly decreased (mesenteric fat, control 1.18 ± 0.069, black tea 1.02 ± 0.094, green tea 1.12 ± 0.07, TF1-water 0.85 ± 0.048 *, total fat, control 5.30 ± 0.35, black tea 4.80 ± 0.26, green tea 5.06 ± 0.36, TF1-water 4.58 ± 0.11 *). In the oral glucose tolerance test in healthy humans, TF1-containing fermentation water (300 mL) (TF1 6 mg, glucose 50 g) significantly suppressed hyperglycemia in a single dose (*n* = 8) and on consumption for 1 (*n* = 6) and 4 weeks (*n* = 5) [single dose: control (glucose 50 g, water 300 mL) 172 ± 5.3, TF1-water 153 ± 18.3 *, placebo 300 mL (glucose 50 g) 172.2 ± 9.1. For 1 week, control 163 ± 19.1; TF1-water 159 ± 28.5; placebo 158.6 ± 27.9. For 4 weeks, start time 187 ± 7.5, TF1-water 161 ± 7.2 **, placebo 189 ± 12.7.

### 8.2. Anti-Periodontitis

Periodontitis is a chronic bacterial infection of supporting structures of the teeth, causing the destruction of both periodontal connective tissues and bone. *Porphyromonas gingivalis*, a Gram-negative anaerobic bacillus, is one of the pathogens involved in the progress of periodontal disease [63], and inflammatory cytokines such as interleukin and tumor necrosis factor in periodontal disease promote periodontal tissue destruction [64,65,66]. On the other hand, matrix metalloprotease (MMP) is known to be a proteolytic enzyme that degrades components of the extracellular matrix including collagen. Another crucial pathogenic effect of *P. gingivalis* is to trigger the activation of MMP-1 (collagenase-1) and MMP-2 (gelatinase-A), as well as interference with the equilibrium between host cell-mediated collagen synthesis and degradation, resulting in irreversible tissue destruction and the progression of periodontitis [67,68].

Zhao et al. reported that black tea extract inhibited the growth of *P. gingivalis* (minimal inhibitory concentrations ranging from 200 to 500 μg/mL; minimal bactericidal concentrations = 500 μg/mL) [69]. In addition, black tea extract dose-dependently inhibited the secretion of IL-6 and IL-8 by *P. gingivalis*-stimulated oral epithelial cells. More specifically, Hosokawa et al. reported that TF3 prevented tumor necrosis factor superfamily 14 (TNFSF 14)-mediated IL-6 production in human gingival fibroblasts. As a mechanism, it was revealed that black tea extract prevented TNFSF 14-induced extracellular signal-regulated kinase, c-Jun N-terminal kinase, and nuclear factor-kB activation in human gingival fibroblasts [19]. These data provide a novel mechanism through which green tea and black tea polyphenols may be used to provide direct benefits in periodontal disease.

Kong et al. investigated the effect of TFs on the pathogenic properties of *P. gingivalis* and on periodontitis by inhibiting matrix metalloproteinases production induced by this oral pathogen [20]. TFs exhibited antimicrobial effects against both planktonic cultures and the biofilm of *P. gingivalis*. TFs also markedly inhibited the proteinase activities of *P. gingivalis* collagenase and gingipains in a dose-dependent manner. Lastly, TFs significantly inhibited the secretion and mRNA expression of MMP-1 and MMP-2 by human gingival fibroblasts stimulated with *P. gingivalis*. TFs appear to also affect the virulent properties of *P. gingivalis* and attenuate the MMP-mediated inflammatory response induced by this pathogen. These findings suggest that TFs may be potentially valuable as supplementary therapeutic agents for the prevention and treatment of *P. gingivalis*-associated periodontal diseases.

### 8.3. Anti-Norovirus

Infections with noroviruses and sapoviruses are a global public health concern because they cause acute gastroenteritis outbreaks in people of all ages, and infections are difficult to prevent and control [70,71]. Currently recommended heat sterilization methods using high temperatures or disinfection methods using sodium hypochlorite cannot be used for the human body such as fingers. For this reason, the establishment of new disinfection methods that can also be used for fingers is required. Ohba et al. conducted cytopathic effect-based screening of 2080 selected compounds to find antiviral compounds against three culturable caliciviruses: feline calicivirus, murine norovirus, and porcine sapovirus [16]. As a result, TF2a, TF2b, and TF3 showed broad antiviral activities against all three caliciviruses. Furthermore, the study of the structure–activity relationship of TFs suggested that the hydroxyl groups of the benzocycloheptenone ring were likely important for the anti-calicivirus activity of TFs. Thus, TFs could be used for calicivirus research and as potential disinfectants and antiviral reagents to prevent and control calicivirus infections in animals and humans.

### 8.4. Anti-Osteoporosis

Osteoporosis, or low bone mineral density, is the single most important risk factor for fracture in older women [72]. Osteoclasts, which are essential in bone homeostasis, play a key role in the development of osteoporosis. Osteoclasts produce MMPs, which play an important role in the degeneration of the matrix associated with bone and cartilage [73]. MMPs comprise a family of zinc-dependent endopeptidases, which includes collagenases (MMP-1, MMP-8, MMP-13, and MMP-18), gelatinases (MMP-2 and MMP-9), and stromelysins (MMP-3 and MMP-10). In particular, MMP-9 is essential with respect to the initiation of the osteoclastic resorption process via the removal of the collagenous layer from the bone surface prior to demineralization [74,75]. It has been reported that older women who drink tea exhibit higher BMD measurements than women who do not drink tea [76]. Oka et al. examined the ability of TF3 and EGCG to suppress individual MMP-2 and MMP-9 enzymatic activity [21]. TF3 or EGCG (10 and 100 µM) were added to cultures of rat osteoclast precursor cells and mature osteoclasts. The numbers of multinucleated osteoclasts and actin rings decreased in polyphenol-treated cultures relative to control cultures. MMP-2 and MMP-9 activities were lower in TF3-treated and EGCG-treated rat osteoclast precursor cells than in control cultures. MMP-9 mRNA levels also declined significantly in TF3-treated osteoclasts in comparison with control osteoclasts. TF3 and EGCG inhibited the formation and differentiation of osteoclasts via inhibition of MMPs. It was revealed that TF3 may suppress actin ring formation more effectively than EGCG.

Metabolic reprogramming occurs in response to the cellular environment to mediate differentiation, but the fundamental mechanisms linking metabolic processes to differentiation programs remain to be elucidated [77]. On the other hand, Nishikawa et al. performed comprehensive metabolomic analysis of osteoclasts and searched for metabolites that increase with osteoclast differentiation [22]. As a result, they found that S-adenosylmethionine (SAM), a metabolite of methionine, was elevated. Furthermore, de novo DNA methyltransferase 3a (Dnmt 3a) was identified as the target molecule of SAM. Nishikawa et al. also found that SAM-mediated DNA methylation by Dnmt 3a regulates osteoclastogenesis via the epigenetic repression of anti-osteoclastogenic genes. The importance of Dnmt 3a in bone homeostasis was further underscored by the observations that *Dnmt3a*-deficient osteoclast precursor cells do not differentiate efficiently into osteoclasts and that mice with an osteoclast-specific deficiency in *Dnmt3a* have elevated bone mass because of a smaller number of osteoclasts. Furthermore, the inhibition of DNA methylation by TF3 has been shown to abrogate bone loss in models of osteoporosis.

## 9. Conclusions

TFs are only present in low concentration in black tea. Therefore, functional studies have lagged seriously behind those of the catechins. Recently, several biosynthetic methods have been developed for the mass production of TFs. Using these methods, further studies are warranted to elucidate the underlying mechanisms of TFs.
